# Novel and holistic approaches are required to realize allelopathic potential for weed management

**DOI:** 10.1002/ece3.10018

**Published:** 2023-04-21

**Authors:** Darwin T. Hickman, David Comont, Amanda Rasmussen, Michael A. Birkett

**Affiliations:** ^1^ Protecting Crops and the Environment Rothamsted Research Harpenden UK; ^2^ School of Biosciences University of Nottingham Sutton Bonington UK

**Keywords:** agriculture, allelochemical, allelopathy, bioherbicide, mode of action, plant defense, weed management

## Abstract

Allelopathy, that is, plant–plant inhibition via the release of secondary metabolites into the environment, has potential for the management of weeds by circumventing herbicide resistance. However, mechanisms underpinning allelopathy are notoriously difficult to elucidate, hindering real‐world application either in the form of commercial bioherbicides or allelopathic crops. Such limited application is exemplified by evidence of limited knowledge of the potential benefits of allelopathy among end users. Here, we examine potential applications of this phenomenon, paying attention to novel approaches and influential factors requiring greater consideration, with the intention of improving the reputation and uptake of allelopathy. Avenues to facilitate more effective allelochemical discovery are also considered, with a view to stimulating the identification of new compounds and allelopathic species. *Synthesis and Applications:* We conclude that tackling increasing weed pressure on agricultural productivity would benefit from greater integration of the phenomenon of allelopathy, which in turn would be greatly served by a multi‐disciplinary and exhaustive approach, not just through more effective isolation of the interactions involved, but also through greater consideration of factors which may influence them in the field, facilitating optimization of their benefits for weed management.

## Graphical abstract

Allelopathy holds promise as an alternative method for weed management, but its application is hampered by limited methodological development and ecological context. This paper therefore considers ecological and evolutionary context for the phenomenon which can aid its further discovery and development, before exploring more holistic approaches and underutilized applications which could benefit agricultural weed management.

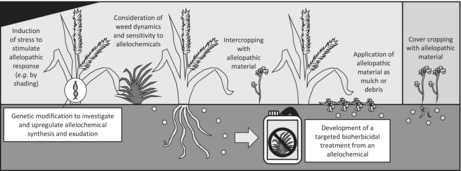

## 1 ALLELOPATHY: AN ENDURING RIDDLE

### 1.1 Allelopathy and common limitations to its examination

**FIGURE 1:**
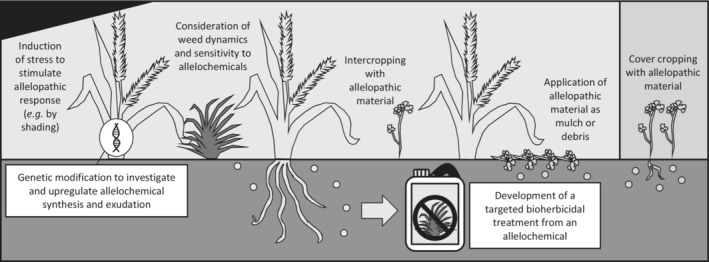
A summary diagram of potential applications for allelopathy in agricultural weed management.

Allelopathy is defined at its broadest as interference between organisms facilitated by secondary metabolites (Mallik & Inderjit, 2002). This usually describes plant–plant interactions, as its original description by Molisch (1937) suggests: ‘The influence of one plant on another’. Given the prevalence and detriment of weeds (Oerke, 2005), and the burgeoning development of populations with resistance to multiple synthetic herbicides (Powles & Yu, 2010), allelopathy may be a valuable alternative to traditional chemical control (Macias et al., 2007), in various forms of delivery, including the deployment of allelopathic crops, intercrops, cover crops, mulches, or bioherbicides derived from plant material (Scavo & Mauromicale, 2021) (Figure 1). Despite its extensive history, however, allelopathy remains underutilized for weed management (Willis, 1985, 2007); underpinning this poor adoption is enduring doubt over the consistency and applicability of allelopathy, based on the inherent difficulty of proving the interaction, and prevailing limitations in experimental practices.

Many allelopathy studies have prioritized approaches which do not adequately consider natural processes or concentrations of putative allelochemicals, such as the extraction of compounds from ground plant tissue, or the identification of phytotoxic doses without consideration of their likelihood in nature (Duke, 2015; Inderjit & Dakshini, 1995; Romeo, 2000). A recent review has furthermore identified that in‐field application of allelopathy for agricultural benefit is commonly undermined by inadequate effort in distinguishing this phenomenon from resource competition in field studies, given the substantial complexity of the system and the similar symptoms that these two interactions produce (Mahé et al., 2022).

Such adherence to flawed or oversimplified approaches in the face of the complicated challenge that weeds pose has proliferated the reputation of allelopathy as a neck riddle, that is, a phenomenon near impossible to prove, but which is logically impossible not to exist (Harper, 1975; Williamson, 1990). Even the pioneering work of Muller (1966) followed frustrated reports of difficulty in elucidating allelopathic interference in desert shrubs; they had previously concluded that even in the relative simplicity of a desert environment, ecological interactions were too complex for allelopathy to be successfully untangled at the time (Muller, 1953). This complexity is further exemplified by the debate stimulated by Muller's reports of allelopathy (Muller, 1966), with Bartholomew (1970) arguing that the observed patterns were attributable to animal consumption. Enduring debate over specific instances of allelopathic potential indicates that such complexity cannot be comprehensively unpicked 70 years later. Such factors, and the range of inhibitory potential that they confer in the field, must therefore be better understood.

Given the complexity of these interactions, then, it is confounding to the understanding of allelopathy that there rarely exists a single study covering in vitro bioassay of crude root exudates, chemical analyses of their composition, the maintenance of effects in biologically active media, and larger‐scale field trials (Inderjit & Nilsen, 2003). This can be partially attributed to a lack of interdisciplinary science (such work covers synthetic and analytical chemistry, plant physiology, soil science, agroecology and agronomy at least, and preferably also genetics, as we will discuss). The exhaustive, multi‐disciplinary work to develop weed‐suppressive rice cultivars is a striking example (Olofsdotter, 2001). Romeo (2000) suggested that the lack of such multi‐disciplinary studies is also a symptom of the modern need for frequent publication in science, noting that early works from times when publication pressure was less severe combined these approaches more effectively (e.g., Muller, 1966).

### 1.2 Enduring doubts lead to limited application or adoption

There is an urgent need for novel and effective approaches for allelopathy to reach application, as its enduring reputation as an almost unknowable quantity has translated to skepticism in end users. The result is a lack of commercialization, adoption, or even knowledge of potential allelopathic benefits in agriculture (Trezzi et al., 2016).

The potential for allelochemicals to contribute to the development of naturally derived herbicides is greatly undervalued. As of 2016, only one commercial bioherbicide was produced from plant extracts (Cordeau et al., 2016), and few others show potential for commercialization (Dayan et al., 2012). Moreover, while allelopathic potential has been identified in many crop species (Worthington & Reberg‐Horton, 2013), breeding for this trait, or even weed suppression more broadly, remains highly atypical. Progress has been most notable in rice, for which constituent allelochemicals and genes involved in their biosynthesis have been identified, and, as a result, particularly allelopathic and weed‐suppressive commercial cultivars have been developed (Belz, 2007; Kong et al., 2011; Olofsdotter, 2001; Serra Serra et al., 2021; Worthington & Reberg‐Horton, 2013).

Even if such allelopathic crops are developed or identified, there is evidence that their potential benefits are not widely known among farmers. Sorghum, through its allelochemical metabolite sorgoleone, represents one of the best understood examples of an allelopathic crop species (Czarnota et al., 2001; Dayan et al., 2010; Głąb et al., 2017; Weston et al., 2013). The allelopathic potential of sorghum may be particularly beneficial given its predominant cultivation in low‐input smallholder systems where both conventional herbicides and labor are not freely available, and therefore options for weed management are limited (e.g., Rodenburg & Johnson, 2009). Additionally, the arid and semi‐arid (and therefore stressful) conditions where sorghum is commonly grown may stimulate allelopathic responses, given their established correlation with abiotic stress (e.g., Tang et al., 1994). Despite this, knowledge of sorghum allelopathy among end users in these systems is limited, with only 29% of farmers in Zimbabwe aware that the species had potential to inhibit weeds in this way (Tibugari et al., 2020). More broadly, only around 10% of rice farmers interviewed in Côte D'Ivoire knew of any potentially beneficial weed‐suppressive plant species (Yao et al., 2019). Knowledge of allelopathy is required for its application to reach a meaningful scale, so these reports emphasize the disconnect between scientific advancements and end‐user understanding.

The widespread uptake of allelopathy‐inspired weed management solutions will only be achieved through reliable approaches to prove and improve their utility, demonstrating their potential efficacy for agricultural weed management. As such, the goal of this paper is to work towards this vision by examining the potential avenues of application for allelopathy (Figure 1), and some approaches within them which we consider to be emergent, novel, or underutilized, and may therefore have the potential to ease the journey from the scientist's laboratory to the farmer's field.

## 2 APPLYING ALLELOPATHY: IMPLEMENTATION IN WEED MANAGEMENT

### 2.1 Asking the right questions: the importance of framework and assay design in allelopathy studies

**FIGURE 2:**
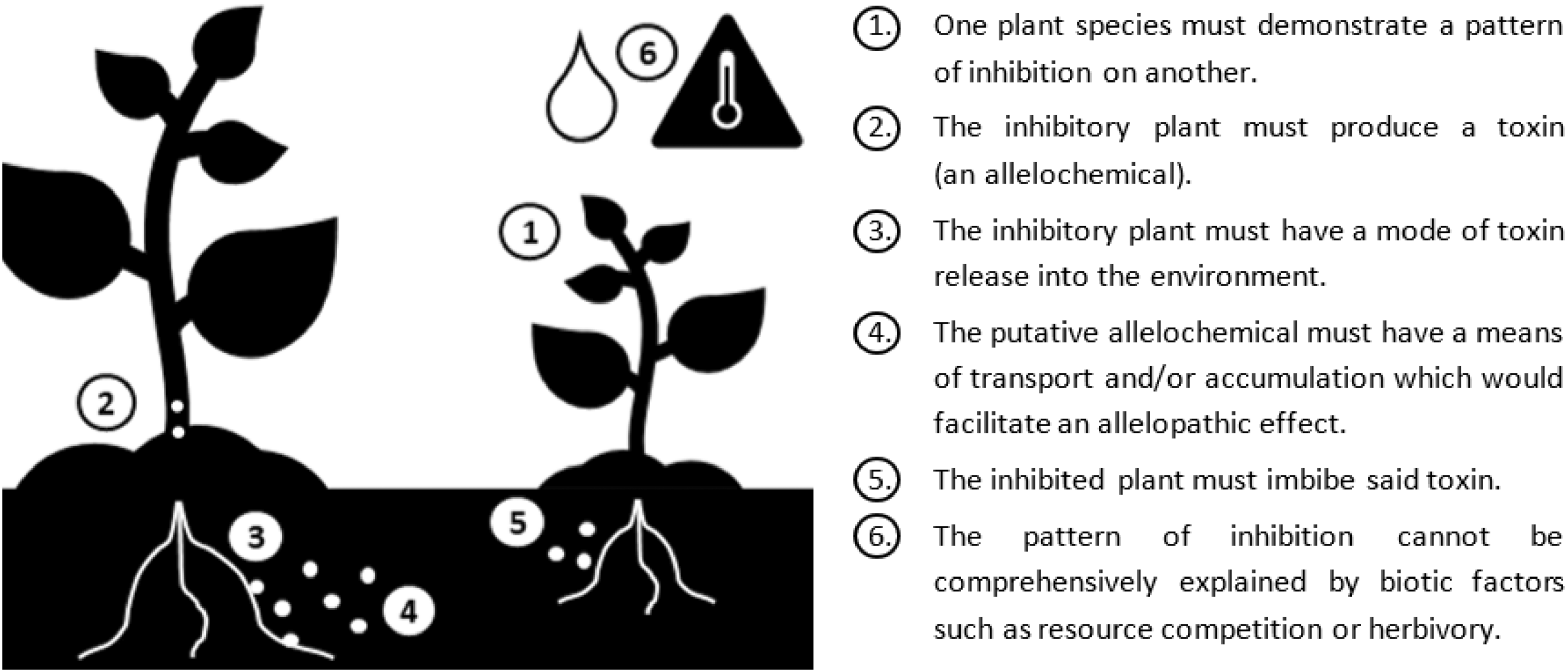
Willis' six points for proving plant–plant allelopathic interactions.

It is logical that effective application of allelopathic material for weed management is predicated on comprehensive elucidation of its effects. As such, studies of allelopathy would benefit from the adoption of an adequate framework from the outset, which seeks to understand the dynamics of allelopathy in each individual case for meaningful application. In perhaps the most comprehensive and specific of these, Willis (1985) offered six points to be satisfied (Figure 2).

Given these questions and the complexities involved in answering them, such frameworks are rarely diligently satisfied, as Willis (1985) had noted when suggesting theirs. This, crucially, is in spite of extensive technological advancements and strides forward in understanding of plant–plant interactions. Thus, while we have novel and increasingly sophisticated tools available to examine allelopathy, they still need to be used to answer the right questions for effective application.

A key aspect of satisfying these frameworks is, of course, the use of the correct assays to elucidate the tenets specified in them. A wide array of systems have been developed and deployed for such purposes, and have been reviewed elsewhere for their strengths and drawbacks (Duke, 2015; Wu et al., 2001), while a number of other works have spotlighted important methodological considerations (e.g., Haugland & Brandsaeter, 1996; Inderjit & Nilsen, 2003). We emphasize that the effective combination of assay methods in both abstract and semi‐natural conditions, focused on different aspects of a suitable framework, is essential for comprehensive understanding of allelopathic phenomena for application. Particularly important is the inclusion of soil as a growth medium in this testing, given the frequent incident of rapid microbial degradation or sorption of putative allelochemicals negating their effectiveness in these conditions (e.g., Kaur et al., 2009).

### 2.2 The challenge of allelochemical discovery: what, and how, can we learn from nature?

The identification of allelochemicals with phytotoxicity to herbicide‐resistant populations may provide a shortcut in novel herbicidal development. The traditional approach of herbicide discovery involves ideation and modeling based on known effective molecules (Peters & Strek, 2018). A comparable, but more ecologically minded approach has recently been advocated in examining the phytotoxic potential of anti‐malarial compounds. This involved the identification of desirable compounds according to the effects of specific functional groups, which were then assessed against *Arabidopsis* seedlings to prove their efficacy, resulting in the successful identification of some candidates for herbicide development (Corral et al., 2017; Sukhoverkov et al., 2021). More generally, the great potential of a modeling approach to identify novel compounds with herbicidal properties is well recognized (Oršolić et al., 2021; Sparks et al., 2017).

It is, however, important to remember the desired end‐goal; a major advantage of naturally derived compounds compared to entirely synthetic alternatives is their perceived environmental safety (Duke et al., 2010). For this reason, modification of a compound found in nature to improve its applicability for weed management carries the risk of producing a less environmentally benign synthetic analog. Still, modeling to identify more effective compounds related to allelochemicals could streamline and assist the development of effective plant‐derived bioherbicides, and improve their chances of commercialization and application.

The existence of bioactive herbicidal compounds also coheres with the theory of multi‐kingdom potential in plant allelochemicals, the extent to which will only be determined by further investigation (Hickman et al., 2021). This theory is further supported by the common identification of traditional medicinal plant species with allelopathic potential, such as olive (*Olea europaea*), squill (*Drimia maritima*), rue (*Ruta graveolens*), lavender (*Lavandula angustifolia*) (Aliotta et al., 2008), sage (*Salvia officinalis*) (Bouajaj et al., 2013), and various mint species (*Mentha* spp., *Nepeta cataria* and *Agastache rugosa*) (Sarheed et al., 2020). Specific allelochemical compounds are also linked with traditional medicines, for example artemisinin (or ‘*Qinghaosu*’) from sweet wormwood (*Artemisia annua*) (Knudsmark Jessing et al., 2014), a traditional treatment for fever in China (Klayman, 1985). This trend has been explored more widely (Islam, Yeasmin, et al., 2018), in broad screens of a large range of species, followed by recommendations of suitable candidates for further work (Fujii et al., 2003; Islam, Hasan, et al., 2018; Sothearith et al., 2021; Suwitchayanon et al., 2017), while other medicinal species have been explored in more detail (Islam & Kato‐Noguchi, 2013). In spite of this work, medicinal plants remain far from commercialization for allelopathic benefit.

Many of the medicinal plants examined for allelopathic potential are of tropical origin (e.g., Sothearith et al., 2021; Suwitchayanon et al., 2017). Ooka and Owens (2018) hypothesized that tropical conditions may be particularly conducive to the evolution of allelopathic potential given otherwise favorable growing conditions and great plant diversity. Thus, exotic plants wherein ‘novel weapons’ to widespread temperate agricultural weeds are more likely to exist may have greater potential for discovery of metabolites with allelopathic properties (Callaway & Ridenour, 2004; Zhang et al., 2021). Searching tropical regions for novel allelopathic plant species would constitute a form of bioprospecting, the search for novel compounds in biodiverse ecosystems, typically for pharmaceutical applications (Mateo et al., 2001). Bioprospecting for agrochemical compounds, although not specifically bioherbicides, has also been advocated (Strobel & Daisy, 2003), and Souza et al. (2008) exemplified this approach in their examination of tropical species from Brazil for control of agricultural pests. Therefore, by extension, plant ecology may provide clues to the identification of new allelopathic plant species, and novel allelochemicals.

### 2.3 Allelopathy‐inspired bioherbicides and modes of action

One major benefit conferred by novel allelochemicals on the route to commercialization is the potential for new, or multiple modes of action to be discovered. Such findings would have potential value in circumventing herbicide resistance, given that this is often conferred by target‐site adaptations (Gressel, 2020; Hachisu, 2021). The discovery of novel herbicide modes of action has been slow since the 1980s, due to widespread reliance on the broad‐spectrum herbicide glyphosate (Dayan, 2019; Duke, 2012; Duke & Dayan, 2022; Peters & Strek, 2018), for which resistance is now prevalent (Heap, 2023; Heap & Duke, 2018). Only one herbicide containing a novel mode of action has been commercialized since this time, the dihydroorotate dehydrogenase inhibitor Tetflupyrolimet (Dayan et al., 2019). Other novel modes of action have been retrospectively identified in old actives (e.g., Cinmethylin and Aclonifen) (Campe et al., 2018; Kahlau et al., 2020), while other recently discovered molecules exhibit novel modes of action which may benefit herbicide development in the future (Shino et al., 2018). Even if an effective and persistent weed inhibitor is identified, however, there remains much outstanding work, examining factors like nontarget toxicity, efficacy toward a wide range of agricultural weeds, and the most effective means of delivery, prior to commercialization.

Even when all of these issues are resolved, it is possible that synthesis of these compounds on sufficiently large scale for field application is prohibitively, or at least antagonistically, expensive (Roberts et al., 2022), owing to their structural complexity and often a lack of preceding work undertaken to optimize their production. Such high production costs of bioherbicides can be an even greater issue if new, cheap, effective ‘traditional’ herbicides are released into the same market; bioherbicidal formulations of the fungus *Colletotrichum gloeosporioides* f.sp. *malvae* for control of *Malva pusilla* were effectively forced out of commercialization in this way in the 1990s (Cordeau et al., 2016). Therefore, while recent progress is encouraging, it is unlikely to be sufficient to outpace the development of herbicide resistance without a change in perspective (Gaines et al., 2021).

It should also be considered that individual modes of action do not exist in a vacuum; identified putative allelochemicals should be examined for interactions (antagonism or synergism) with each other, given potential consequences for application. There is precedent for allelochemicals to synergize and enhance inhibitory effects (e.g., Einhellig & Rasmussen, 1978). Recent work has moreover identified compounds with indirect benefit for weed control, inhibiting the detoxification enzymes of plant species with metabolic (nontarget) herbicide resistance, thereby essentially rendering the plant herbicide‐susceptible (Schwarz et al., 2021). It is therefore logical that plants have evolved to synthesize co‐occurring compounds which synergize to more effectively suppress competing species, but this has not been widely explored at present.

### 2.4 Allelopathic crops and the potential for benefits from understanding of genetics

The deployment of allelopathic crops may be a feasible application of the phenomenon for the management of herbicide‐resistant weeds. This is the case even in the absence of detailed mechanistic understanding, as much as it is desired for the understanding of allelopathic effects and the optimization of their application; the prominence of mechanistic aspects in the frameworks suggested earlier in this piece exemplify their importance. Such application would benefit from contributions to the emergent understanding of plant–plant communication and recognition (discussed later in this piece), as well as greater understanding of potential nontarget effects of putative allelochemicals (Fritz & Braun, 2006). Such understanding would elevate allelopathic plants from blunt objects for weed control into intelligent tools to fit into an integrated weed management program.

A major detrimental factor in the development of allelopathic cash crops (e.g., Table 1) is the demand of yield. Agriculture has long prioritized breeding for yield improvement over other traits, so any form of weed suppression should consider net effect on productivity, given that reduced yield in a weed‐free environment can be compensated by the yield benefit provided by effective weed suppression (Worthington & Reberg‐Horton, 2013). This is exemplified by the breeding of allelopathic rice cultivars by Kong et al. (2011). While this work proves that a high‐yielding, weed‐suppressive crop variety can be achieved, much effort is required to characterize the trade‐offs related to yield and plant defense.

**TABLE 1:** Common crop species where allelochemicals and their effects have been substantiated and reviewed.

Crop species	Major allelochemicals	Summary references
Rice	Momilactones A and B	Serra Serra et al. (2021)
Sorghum	Sorgoleone	Głąb et al. (2017), Weston et al. (2013)
Wheat, Rye, Maize	Benzoxazinoids (DIMBOA, DIBOA)	Wouters et al. (2016)
Brassicas	Glucosinolates (isothiocyanates)	Rehman et al. (2018)
Barley	Hordenine, Gramine	Jabran (2017)

A sophisticated, yet largely underappreciated approach in developing allelopathic crops for weed management is through application of genetic engineering techniques to better understand and deploy them (Aci et al., 2022). In sorghum, recent effort has been made to identify genetic regions involved in sorgoleone biosynthesis (Shehzad & Okuno, 2020), paving the way for future efforts to upregulate these genes for greater allelopathic effect. There is, interestingly, evidence that cytochrome P‐450 monooxygenases (which have a range of functions in stress responses) play a role in sorgoleone synthesis (Pan et al., 2018), as well as the biosynthesis of other allelochemicals in other plant species (Serra Serra et al., 2021), indicating some consistency between species in their genetic tools for allelochemical synthesis.

A related option is to alter the genes coding for biosynthesis of the identified allelochemical, for instance through knock‐down (Yoshida et al., 2017), theoretically facilitating comparison with a wild type for allelopathic effects. Again, these genes could also be upregulated for greater synthesis or exudation of potent allelochemicals. Promising advances in gene editing technology suggest potential in modifying wheat gene expression to alter desirable traits (Zhang et al., 2016), for example in the editing of an asparagine synthetase gene to produce wheat with reduced content of free asparagine (Raffan et al., 2021). Similar methods could edit specific genes involved in benzoxazinoid allelochemical biosynthesis in cereals (Frey et al., 1997; Makowska et al., 2015; von Rad et al., 2002), allowing the examination or upregulation of specific compounds in the pathway.

Genetic modification for study of allelopathy could be hindered by the possible effects of gene modification on plant growth (and therefore ability to compete for resources). Züst et al. (2011) reported that the removal of allelochemical synthesis genes confirmed the fitness cost of defense metabolite synthesis, as the knockout of glucosinolate biosynthesis genes in *Arabidopsis thaliana* stimulated early growth in mutants compared to the more defensively capable wild‐type *Arabidopsis* plants examined. Nonetheless, with understanding of such effects, the editing of allelochemical synthesis genes may be a useful tool for the examination of the effects of allelopathy.

### 2.5 Cover cropping and intercropping: allelopathy as a benefit

Allelopathic species could alternatively be applied as cover or intercrops for weed suppression, in rotation or sown with a less weed‐suppressive cash crop (Jabran et al., 2015). Cereals such as rye have been advocated in this manner, given their well‐substantiated allelopathic potential (Masiunas et al., 1995; Rice et al., 2012), while brassicaceous species, exuding glucosinolate allelochemicals (Al‐Khatib et al., 1997; Haramoto & Gallandt, 2004; Rehman et al., 2018), and a number of legumes (Adler & Chase, 2007; White et al., 1989), have also been suggested to be allelopathic. Importantly, however, legumes also exemplify a potential complication in applying such material in weed management, given that these plants also fix nitrogen. This is obviously an additional benefit for the crop in the system, but it should be noted that some weed species may respond positively to this resource input (Jäck et al., 2021), nullifying their inhibition by allelochemicals (or, for that matter, resource competition). For this reason, it is essential to understand the system that these applications will enter into, given the consequences that this may have for the outcome.

Common in these effective allelopathic cropping schemes is the use of decomposing plant tissues after growth (e.g., Al‐Khatib et al., 1997; White et al., 1989). Previous works have specifically noted the allelopathic potential of wheat straw (Al Hamdi et al., 2001; Steinsiek et al., 1982), and rye mulch (Tabaglio et al., 2008) on weeds. Given that benzoxazinoid content within plant tissues is likely to be greater than levels in root exudate (Escobar & Niemeyer, 1993; Hussain et al., 2022; Stochmal et al., 2006), the application of cereal debris would increase allelochemical concentration and also delay its release as tissues decompose, potentially delaying degradation of these short‐lived compounds (e.g., Macías et al., 2004).

An intriguing potential modification of these systems is to use crop or cultivar mixtures rather than a single uniform biotype. There is evidence of weed suppression being increased through mixing of species or cultivars (Baraibar et al., 2017; Smith et al., 2020), which is likely to be attributable to both resource competition and allelopathy, as plants are known to detect and modify their responses to neighbors in terms of both of these phenomena (Dudley et al., 2013; Yang et al., 2018). This can be highly variable even between biotypes of the same species. However, Xu et al. (2021) reported that relatively closely related rice cultivars can be more effective in competitive suppression of paddy weeds in spite of reduced allelochemical exudation, while another recent work appears to suggest that plants more functionally similar to the allelopathic species (and therefore potentially perceived as ‘more of a threat’ to their niche) will stimulate a stronger allelopathic response, at least in the case of wheat exuding benzoxazinoids (Hussain et al., 2022). Such findings indicate that the correct combination of crops or even cultivars may have potential to stimulate and maximize weed suppression through allelopathy due to these recognition interactions.

**FIGURE 3:**
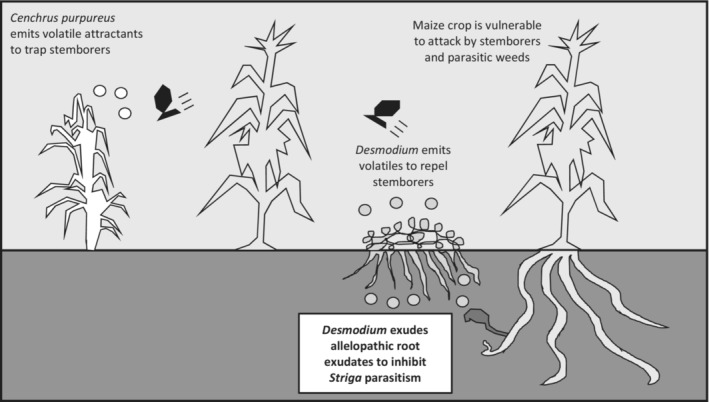
A summary of the push–pull system in maize, with the allelopathic element labeled in bold. Adapted from Khan et al. (2014).

Arguably the most complete and successful application of an allelopathic intercrop is the leguminous genus *Desmodium* (most notably *D. uncinatum* and *D. intortum*), deployed in smallholder maize systems in Africa. *Desmodium* is highly suppressive to parasitic *Striga* spp. through allelopathic action (Khan et al., 2002), while also being repulsive to stemborer species like *Chilo partellus* and *Busseola fusca*, thereby bringing additional defensive benefit to the crop (Khan et al., 2006) as a constituent of a so‐called push–pull system (Khan et al., 2014) (Figure 3). This technology is now reaching widespread application in these areas (uptake of over 200,000 farmers), albeit with alterations dependent on the specific requirements of the individual farmer (Pickett & Khan, 2016), and should therefore be considered an example for successful incorporation of allelopathic potential into cover or intercrop systems.

### 2.6 Plant stress and allelopathy: using it to our advantage

Given the well‐established link between stress and allelopathy (Reigosa et al., 1999; Tang et al., 1994), the induction of mild stress can be an elicitor of allelochemical exudation in a manner which may be of net benefit for weed management. This may be especially valuable because such stresses have potential to simultaneously increase the vulnerability of nearby target weeds to these allelochemicals. Scavo et al. (2020) demonstrated that anthropogenic shading of cardoon (*Cynara cardunculus*) led to increased concentrations of phytotoxic sesquiterpene lactones, suggesting the potential of this approach, although, of course, it does carry the danger of unintended detriment to the allelopathic species if the stress is too intense.

A sophisticated alternative approach for maximizing allelochemical delivery to an intended target lies in the application of additional compounds with indirect effects. The exogenous application of stress signaling compounds like *cis*‐jasmone and jasmonic acid has stimulated benzoxazinoid accumulation in wheat tissues (Moraes et al., 2008; Sue et al., 2021), sorgoleone accumulation in sorghum (Uddin et al., 2013), and comparable defensive responses in other crop species like rice (Bi et al., 2007), and potato (*Solanum tuberosum*) (Sobhy et al., 2017). This may present a method for augmenting allelopathic exudation where desired. The induction of polyploidy in wheat, rye, and maize with colchicine in vitro has also been correlated with increased benzoxazinoid exudation, given the existence of more genetic material conferring it (Oliveros‐Bastidas et al., 2018). The application of this finding in‐field would require extensive work to ensure that other developmental metrics are not detrimentally affected, but at the least, it vindicates the examination of inducibility interactions and, more broadly, approaches to stimulate allelochemical exudation for weed management.

### 2.7 Know your enemy: understanding the target to optimize allelopathic inhibition

As noted in previous sections, it is fundamental to application of allelopathic material for weed management to consider the intended target weed. Such species have too often been viewed as a homogeneous enemy to be overcome, rather than living, interacting, evolving species in a wider agroecological environment (Neve et al., 2009); the proliferation of herbicide resistance highlights the danger of this approach (Powles & Yu, 2010). Although outside of the scope of this review, it should furthermore be noted that such weeds are in some cases allelopathic themselves (Guo et al., 2017), which obviously has potential to alter such crop–weed interactions. Key to the greater application of allelopathy in weed management is in a more integrated approach, with greater consideration of both the ‘donor’ and ‘target’ plant species, and the dynamics within an agroecosystem which modulate their interactions (Cheng & Cheng, 2015).

As such, allelochemical sensitivity in target species is highly variable, and dependent on specific factors. For example, it is commonly observed that plant roots are more sensitive to allelochemicals than plant aboveground tissues (Haugland & Brandsaeter, 1996), and that older plants in general display reduced allelochemical sensitivity (Zhang et al., 2021). Taken in isolation, it could be construed that allelopathy therefore is of little consequence for in‐field application, where crop–weed competition is greatest between plants at later developmental stages, and strongly influenced by relative aboveground biomass. Nevertheless, this simplistic interpretation overlooks the fact that inhibition of weed development at an early developmental stage has the potential to profoundly alter later crop–weed competitive dynamics (Storkey et al., 2021). It is therefore important that indirect effects at later developmental stages are not overlooked when evaluating the efficacy of allelopathic applications. It is unreasonable to assume that any control measure could be a “silver bullet” against an herbicide‐resistant weed in the manner expected of an effective synthetic herbicide. Rather, the use of allelopathy can contribute another of the “many little hammers” required for such a program in place of conventional chemical control (Liebman & Gallandt, 1997).

## 3 CONCLUSION: INTEGRATIVE THINKING AS THE KEY TO ALLELOPATHY REACHING APPLICATION

While plant‐derived allelochemicals should never be considered as silver bullets for weed management, either as a component of weed suppression from a potent cash crop, cover crop, or intercrop species, or as directly applied phytotoxins examined for inhibitory potential and mode of action, there is much unrealized potential in allelopathy and the compounds which induce it. It is apparent, however, through review of current understanding of this phenomenon as it pertains to application for weed management, that there is no universally effective means for deploying allelopathy. Rather, its optimal use is dictated by individual scenarios based on preceding knowledge, agricultural management options, the compounds and crops involved, the target weed itself, and other factors relating to the environment. The effort required to effectively develop and apply allelopathy as part of a weed management strategy is substantial, which speaks to the continued reliance of agriculture on easier, but less sustainable short‐term fixes (MacLaren et al., 2020).

While there is knowledge of a large number of allelopathic crops, it is likely that an even larger number of allelopathic plant species exist which are not currently considered economically valuable, but which could provide currently untapped benefits for the agricultural system. This is perhaps a telling indictment on the current status of allelopathy as a viable weed management option, given that allelopathic potential may be agriculturally beneficial in itself, in the same manner as highly competitive cover crops. For this reason, a more holistic and integrative approach is required to elevate allelopathy to widespread application, applying a detailed framework to track the phenomenon in vitro, in the glasshouse, and in the field, and disproving alternative explanations for the inhibitory patterns observed, before tailoring its application to the specific scenario to maximize its effectiveness for weed management.

## AUTHOR CONTRIBUTIONS

**Darwin T. Hickman:** Conceptualization (lead); project administration (supporting); writing – original draft (lead); writing – review and editing (equal). **David Comont:** Conceptualization (supporting); project administration (equal); supervision (equal); writing – review and editing (equal). **Amanda Rasmussen:** Conceptualization (supporting); project administration (equal); supervision (equal); writing – review and editing (equal). **Michael A. Birkett:** Conceptualization (supporting); project administration (lead); supervision (lead); writing – review and editing (equal).

## Data Availability

As this manuscript is a review, data sharing is not applicable to this article as no datasets were generated or analysed.
